# Elongated, Pedunculated Verruca Vulgaris of Both Upper Eyelids: A Rare Bilateral Presentation

**DOI:** 10.7759/cureus.99375

**Published:** 2025-12-16

**Authors:** Yuma Iwasaki, Hiroshi Toshida

**Affiliations:** 1 Ophthalmology, Juntendo University School of Medicine, Bunkyō, JPN; 2 Ophthalmology, Juntendo University Shizuoka Hospital, Izunokuni, JPN; 3 Ophthalmology, St. Marianna University Yokohama Seibu Hospital, Yokohama, JPN

**Keywords:** bilateral involvement, eyelid tumor, hpv, human papillomavirus, pedunculated mass, verruca vulgaris

## Abstract

A 71-year-old woman presented with progressive visual decline due to bilateral upper eyelid masses and bilateral cataracts that obstructed her visual axis. The lesions had gradually elongated over approximately ten years after a dermatologist deemed surgical removal impossible. Excision of the pedunculated tumors was performed prior to cataract surgery because the lesions drooped over the visual axis and would have obstructed the operative field. Subsequent cataract surgery restored visual acuity to 20/20 in the right eye and 20/25 in the left. This case illustrates that verruca vulgaris of the eyelid, though benign, can elongate into large pedunculated masses if neglected, potentially leading to functional visual impairment. As this condition is associated with infection by low-risk human papillomavirus (HPV) types, careful postoperative monitoring for recurrence is required.

## Introduction

Eyelid tumors are among the lesions frequently encountered in daily ophthalmic practice, and most are benign in nature. Patients with benign lesions often seek medical attention only when cosmetic concerns or functional visual disturbance become significant [1-8]. Verruca vulgaris represents only a small subset of benign eyelid lesions and is typically unilateral and small in size. Bilateral involvement is exceedingly rare, and previous reports have not documented cases with such marked elongation of the pedunculated lesions [1-3,6-8]. We report a rare case of verruca vulgaris presenting as unusually long, pedunculated masses on both upper eyelids, which had progressively elongated over approximately 10 years to the point of interfering with the patient’s visual function.

## Case presentation

**Figure 1:**
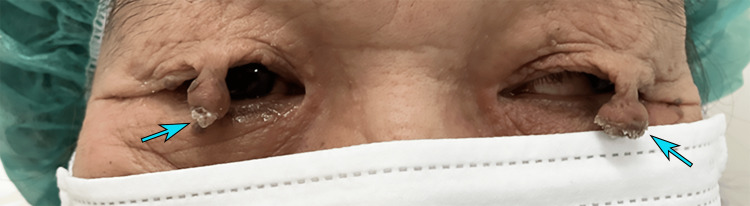
External appearance at initial visit Large, elevated, pedunculated masses are present on both upper eyelids. Blue arrows indicate the distal tips of the lesions, which drooped over the visual axis and contributed to visual obstruction

A 71-year-old woman presented with bilateral visual disturbance. Approximately 10 years earlier, she had noticed nodules on both upper eyelids that had gradually enlarged over time (Figure 1). A dermatologist had previously diagnosed the lesions as benign and informed the patient that surgical removal would be difficult; however, the specific reason for this assessment was not documented. Based on the available information, it remains unclear whether the difficulty was attributed to the lesion size, pedunculated morphology, or other clinical considerations, as no detailed explanation was recorded.

**Figure 2:**
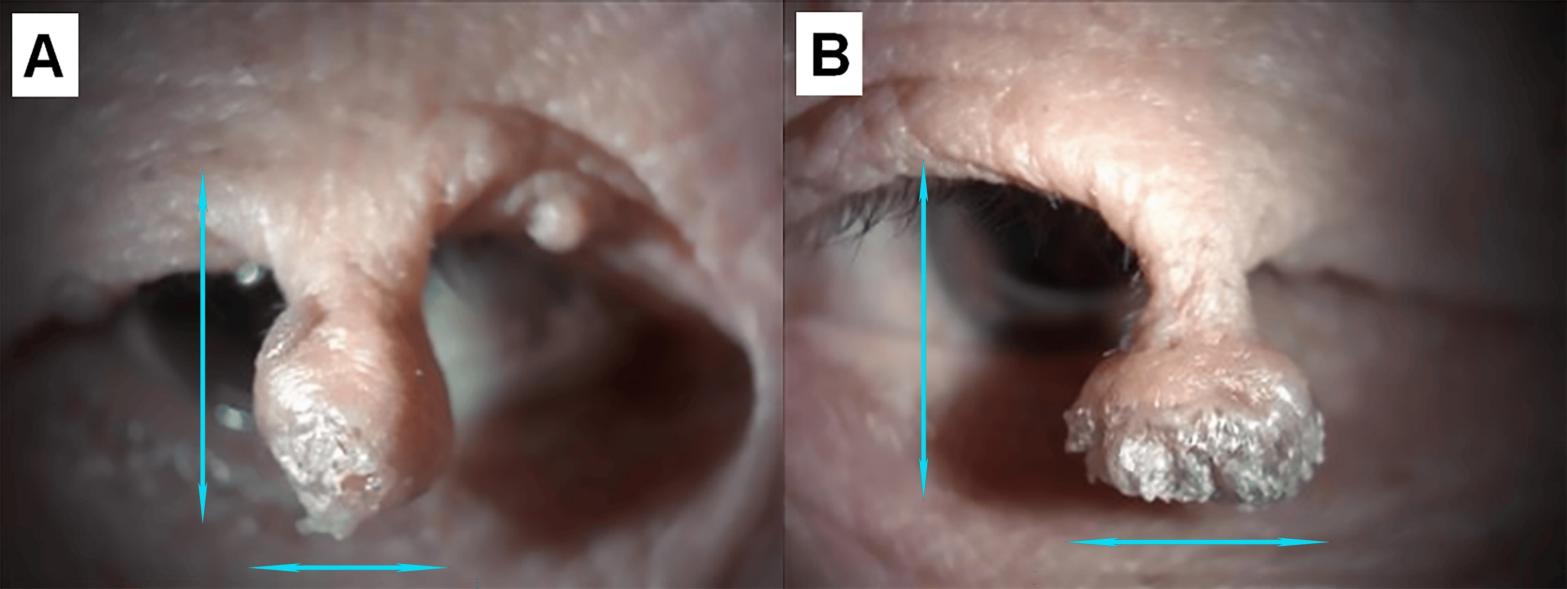
Magnified views of the eyelid masses Close-up photographs of the right (A) and left (B) upper eyelids show elongated pedunculated lesions. Both masses hang down from the upper eyelids and partially obstruct the visual axis. The lesion surfaces exhibit hyperkeratosis, consistent with the typical appearance of verruca vulgaris. Blue arrows indicate the measurement points for each lesion. The right-sided lesion measured 13.5 mm in length with a distal width of 6.5 mm, while the left-sided lesion measured 12 mm in length with a distal width of 9 mm

**Figure 3:**
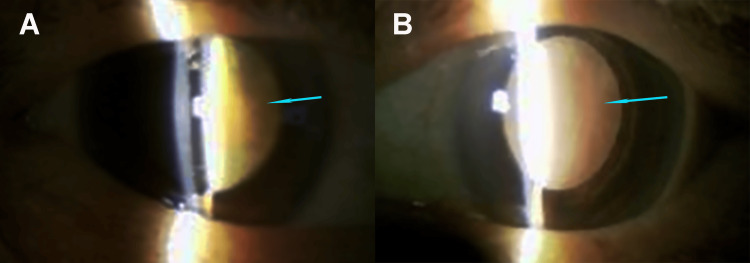
Slit-lamp examination After pupil dilation, slit-lamp examination revealed mature cataracts in both eyes, preventing visualization of the fundus. Blue arrows indicate the dense opacity in each eye. (A) Right eye. (B) Left eye

Her medical history included hypertension and diabetes mellitus. At the initial examination, uncorrected visual acuity (UCVA) was 20/800 in the right eye, which was uncorrectable, and hand motion in the left eye. Slit-lamp examination revealed large pedunculated masses hanging from both upper eyelids (Figure 2) and mature cataracts in both eyes (Figure 3). Refraction could not be performed because the elongated eyelid masses partially obstructed the visual axis, limiting accurate measurement.

The mass on the right upper eyelid measured 13.5 mm in length, with a distal width of 6.5 mm and a basal width of 5 mm. The mass on the left upper eyelid measured 12 mm in length, with a distal width of 9 mm and a basal width of 3 mm. Neither lesion involved the eyelid margin or eyelashes. Standard eyelid measurements, such as the margin reflex distance 1 (MRD1), could not be reliably obtained because the elongated pedunculated masses completely covered the central visual axis. Formal visual field testing was also not feasible for the same reason, and the obstruction was documented clinically based on direct observation. Fundus examination was not possible because of lens opacity. B-scan ultrasonography was performed and showed no vitreoretinal abnormalities.

Although cataract surgery was indicated, the drooping eyelid masses interfered with the visual axis and would have hindered the procedure. Therefore, excision of the eyelid tumors was performed first. Because the surrounding eyelid skin had sufficient laxity, complete surgical excision with an adequate safety margin was possible. The excision was performed under local anesthesia according to standard oculoplastic principles. A 2-mm safety margin was chosen to ensure complete removal in case occult malignancy could not be fully excluded preoperatively. An approximately 2-mm safety margin was then marked around the tumor base, and the lesion was resected using a clamp-assisted technique to minimize bleeding. The wound was closed with three interrupted 6-0 nylon sutures. Because the depth of involvement could not be determined preoperatively, excision of the right eyelid lesion was performed first. Once intraoperative findings confirmed that the tumor was confined to the skin without deeper tissue involvement, excision of the left eyelid lesion was safely performed the following day. The main procedural steps are summarized in Table 1.

**Table 1:** Summary of the surgical procedure

Step	Key surgical action	Purpose/note
1	Measure tumor size and mark resection line with a 2-mm safety margin	Ensure complete removal with minimal tissue loss
2	Inject 1% lidocaine with epinephrine subcutaneously	Local anesthesia and hemostasis
3	Apply eyelid clamp to the tumor pedicle	Stabilize lesion and reduce bleeding
4	Incise skin along the marked line using a scalpel	Access tumor base
5	Excise tumor using spring scissors	Remove lesion completely
6	Achieve hemostasis and close wound with three interrupted 6-0 nylon sutures	Restore eyelid contour
7	Apply topical antibiotic ointment	Postoperative wound care

**Figure 4:**
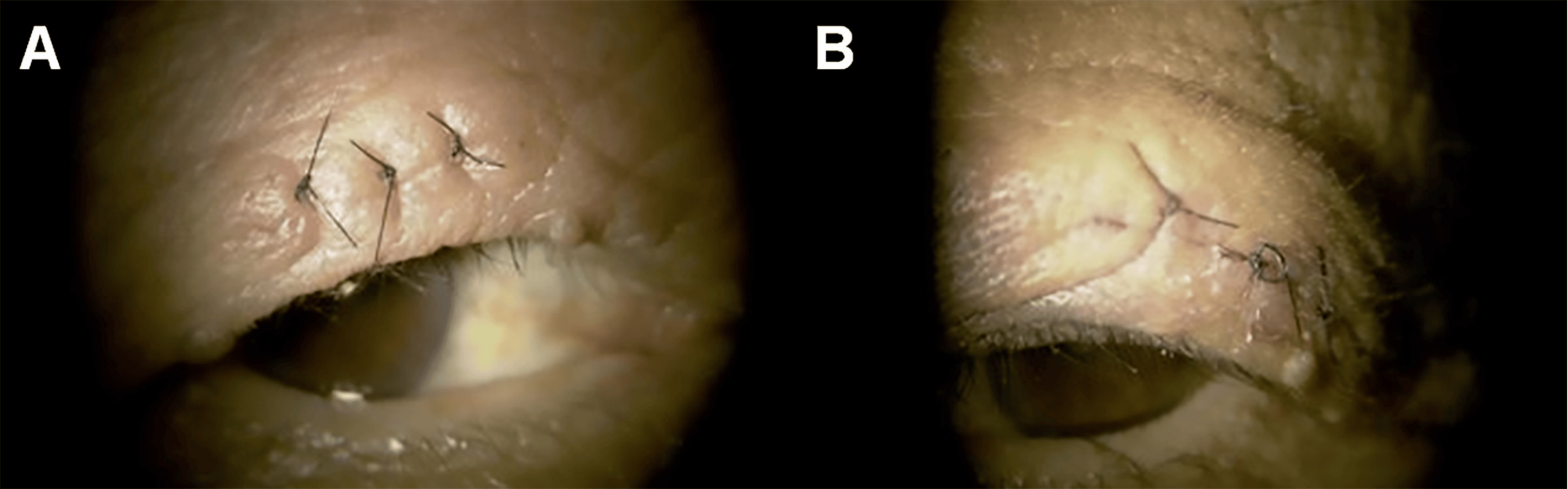
One week after sequential bilateral eyelid tumor excisions External photographs taken one week postoperatively demonstrate good wound healing in the right upper eyelid (A) and left upper eyelid (B). Histopathological examination confirmed the diagnosis of verruca vulgaris, and cataract surgery was subsequently planned. Both eyes exhibited mature cataracts, and lens reconstruction surgery was scheduled as the next step. (A) Right eye. (B) Left eye

The postoperative course was uneventful (Figure 4).

**Figure 5:**
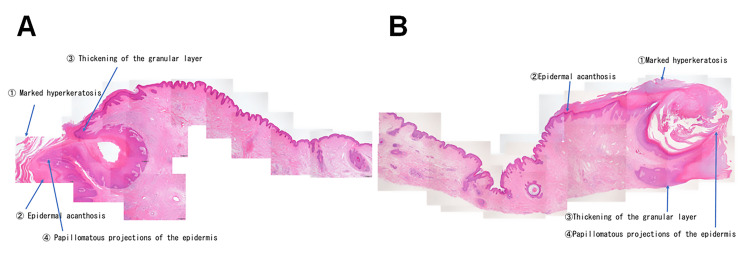
Histopathological Findings (hematoxylin-eosin staining) Microscopic examination of the excised specimens from both eyelids revealed similar histopathological features, including marked hyperkeratosis, acanthosis, thickening of the granular layer, and epidermal proliferation consistent with verruca vulgaris. (A) Right upper eyelid. (B) Left upper eyelid

Histopathological examination of the excised specimens from both eyelids revealed marked hyperkeratosis, acanthosis, thickening of the granular layer, and papillomatous epidermal proliferation, findings consistent with a diagnosis of verruca vulgaris (Figure 5).

**Figure 6:**
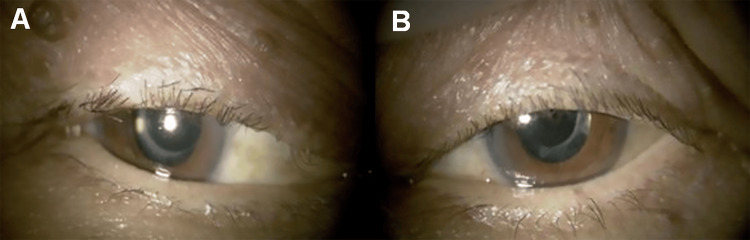
Three months after sequential eyelid tumor excisions and subsequent cataract surgeries Postoperative photographs taken three months after sequential eyelid tumor excisions followed by cataract surgeries. Excellent wound healing and symmetrical eyelid contour are observed. Visual acuity improved as a combined result of removing the pedunculated eyelid masses, thereby clearing the visual axis, and subsequent cataract extraction. (A) Right eye. (B) Left eye

Although human papillomavirus (HPV) genotyping was attempted, the currently available commercial assays in Japan do not include low-risk HPV subtypes such as HPV-2, -27, and -57; therefore, no detectable subtype was identified. Subsequently, phacoemulsification with intraocular lens implantation was performed in both eyes. Three months after cataract surgery, the best-corrected visual acuity improved to 20/20 (logMAR 0.00) in the right eye and 20/25 (logMAR 0.10) in the left eye (Figure 6). This improvement reflected both the removal of the eyelid masses, which eliminated obstruction of the visual axis, and the subsequent cataract extraction.

The patient was followed for 12 months after surgery, with examinations performed at one week, one month, three months, six months, and 12 months. At each visit, eyelid margin morphology, the presence of new papillomatous lesions, wound healing status, and overall eyelid contour were assessed using slit-lamp examination and photographic documentation. No recurrence or new lesions were observed during the follow-up period. Given the association of verruca vulgaris with low-risk HPV types, continued long-term monitoring beyond the first postoperative year is recommended to detect any potential recurrence. A video demonstrating the surgical procedure has been uploaded as supplementary material (Video 1).

**Video 1:** Surgical excision of bilateral pedunculated verruca vulgaris of the upper eyelids This video demonstrates clamp-assisted excision of pedunculated verruca vulgaris on both upper eyelids under local anesthesia, with a 2-mm safety margin marked around the tumor base and closure using interrupted 6-0 nylon sutures

## Discussion

Differential diagnoses for pedunculated eyelid lesions include squamous papilloma, seborrheic keratosis, pedunculated chalazion, cystic lesions, and, less commonly, malignant tumors such as squamous cell carcinoma or basal cell carcinoma with exophytic growth. In the present case, the lesions were long-standing, bilaterally symmetrical, and slow-growing and exhibited a hyperkeratotic verrucous surface without ulceration or induration, making malignancy unlikely. Histopathological examination demonstrated characteristic features of verruca vulgaris-marked hyperkeratosis, acanthosis, and papillomatous proliferation, thereby definitively excluding these differential diagnoses.

Previous studies have reported that verruca vulgaris accounts for approximately 3.8-5.7% of benign eyelid tumors in Japan [1,6]. However, nearly all documented cases involve unilateral lesions, and bilateral involvement of eyelid verruca vulgaris is exceedingly rare. To our knowledge, no clearly documented cases of bilateral eyelid verruca vulgaris have been reported in the literature. Therefore, the present case, with markedly elongated, pedunculated lesions occurring simultaneously on both upper eyelids, appears to represent an exceptionally rare clinical presentation. Furthermore, none of the previously reported cases described lesions with such pronounced, snail-horn-like elongation or with a similarly prolonged course over approximately 10 years.

As ophthalmologists, we are not accustomed to encountering verruca vulgaris, which is primarily managed by dermatologists. In this case, the lesions were left untreated for approximately 10 years after a dermatologist deemed surgical removal difficult; however, no explanation was documented. Based on the lesion size, marked elongation, and pedunculated morphology observed at presentation, it is plausible that the dermatologist judged the lesions to fall outside the practical indications for first-line treatments such as cryotherapy or salicylic acid application. According to the Japanese Dermatological Association guidelines, these modalities are recommended for small, localized lesions (grades A and B), whereas surgical excision is classified as a grade C1 (“may be considered”) option for larger or atypical lesions. In this case, the substantial tumor size and elongated pedicles made excision the most appropriate approach [9]. Similar guidance is reflected in the British Association of Dermatologists’ guidelines, which likewise emphasize cryotherapy and salicylic acid as first-line treatments [10].

The causative agents of verruca vulgaris are low-risk HPV subtypes, most commonly HPV-2, HPV-27, and HPV-57, which are known to cause occasional recurrences. These HPV types have also been identified in benign eyelid lesions, supporting the viral etiology reported in previous studies. HPV typing was attempted in the present case; however, currently available commercial assays in Japan no longer include these low-risk subtypes, and therefore, the causative subtype could not be identified. The diagnosis was established based on the characteristic clinical morphology and histopathological findings [10,11]. Because verruca vulgaris may recur due to its viral origin, careful long-term follow-up is recommended. In this patient, examinations were performed at one week, one month, three months, six months, and 12 months after surgery, with evaluation of eyelid margins using slit-lamp examination and photographic documentation. No recurrence was observed during the 12-month period.

Even benign eyelid tumors can progressively enlarge over time and lead to functional impairment if left untreated [1]. This case demonstrates that verruca vulgaris of the eyelid, when neglected, may develop into large pedunculated masses capable of obstructing vision. The bilateral, snail-horn-like appearance of the lesions was particularly distinctive, and to our knowledge, no previously published reports have described a similar bilateral presentation. This underscores the importance of early evaluation and appropriate interdisciplinary management.

## Conclusions

We reported a rare case of bilateral pedunculated masses on the upper eyelids. Surgical excision was performed because the lesions obstructed the patient’s visual axis and interfered with cataract surgery. Histopathological examination confirmed the diagnosis of verruca vulgaris. As this condition is associated with infection by low-risk HPV types, careful postoperative monitoring is required to detect possible recurrence. This case highlights that even benign eyelid lesions, when left untreated, can enlarge sufficiently to cause functional visual impairment. This underscores the importance of timely evaluation as well as coordinated interdisciplinary management, both in determining the optimal timing of surgical intervention and in ensuring appropriate follow-up, between ophthalmologists and dermatologists.
